# Using Volatile Oxidation Products to Predict the Inflammatory Capacity of Oxidized Methyl Linoleate

**DOI:** 10.3390/foods14244231

**Published:** 2025-12-09

**Authors:** Zhiwen Zhang, Luocheng Zhang, Xinxin Jiao, Sasa Zhao, Hua Wu, Junsong Xiao

**Affiliations:** 1 School of Food and Health, Beijing Technology and Business University, Higher Education Garden, Liangxiang, Fangshan District, Beijing 102488, China; zhangzhiwen0604@163.com (Z.Z.); zhanglc2060@163.com (L.Z.); zhaosasa2022@163.com (S.Z.); 2 School of Light Industry Science ang Engineering, Beijing Technology and Business University, Higher Education Garden, Liangxiang, Fangshan District, Beijing 102488, China; jiaoxx0822@163.com (X.J.); wuhua@btbu.edu.cn (H.W.)

**Keywords:** lipid oxidation, methyl linoleate, HS-SPME-GC-MS, RAW264.7 cells, inflammation, model prediction

## Abstract

This study evaluated whether the volatile profile of methyl linoleate (MLO) can predict its pro-inflammatory capacity. MLO was subjected to two oxidation conditions simulating ambient storage and high-temperature frying. Free radicals, volatile compounds, and aldehydes were quantified using ESR, HS-SPME-GC-MS, and UPLC-MS/MS. Oxidized MLO was applied to RAW264.7 macrophages to evaluate inflammatory cytokines and oxidative stress responses, and PLSR models were developed to predict cellular outcomes based on volatile fingerprints. Both oxidation conditions induced substantial increases in short-chain and unsaturated aldehydes, with high-temperature oxidation generating markedly higher levels of key volatiles. Oxidized MLO significantly elevated TNF-α, IL-1β, COX-2, ROS, NO, and MDA while reducing SOD activity (*p* < 0.05), demonstrating strong pro-inflammatory and pro-oxidant effects. Volatile-based PLSR models achieved high predictive performance, with cross-validated and external R^2^ values approaching 0.9 and RPD values exceeding 2. These findings show that volatile oxidation products reliably reflect the pro-inflammatory potency of oxidized lipids and can support the ranking of oxidized oils and lipid-rich foods, as well as guide processing and dietary strategies.

## 1. Introduction

Linoleic acid (C18:2), an ω-6 polyunsaturated fatty acid, is abundant in edible oils and serves as both a structural lipid and a precursor of bioactive eicosanoids [1]. In processed foods, however, thermal treatments and prolonged storage accelerate lipid peroxidation via free-radical chain reactions and generate primary and secondary oxidation products [2]. These products are highly reactive and can compromise food safety. In particular, secondary aldehydes such as malondialdehyde (MDA) and 4-hydroxy-2-nonenal (4-HNE) have been implicated in inflammatory signaling and immune dysregulation, thereby contributing to the pathogenesis of cardiometabolic disorders [3]. Linoleic acid constitutes approximately 40% of total fatty acids in common vegetable oils and 70–85% in certain oilseeds [4,5]. A thorough understanding of its oxidative transformation pathways and health effects is therefore critical for the rigorous evaluation of edible-oil quality and safety. Given that unsaturated fatty acyl chains govern the oxidation chemistry of oils and that most fatty acids in plant oils are esterified, fatty-acid methyl esters (FAMEs) provide a practical, compositionally simple, mechanism-informed surrogate for modeling edible-oil constituents [6].

In recent years, multivariate chemometric approaches—such as partial least squares regression (PLSR)—have been widely used to relate analytical measurements to indices of oxidation and risk, owing to their straightforward implementation and strong predictive performance. Conventional assessments of oil deterioration typically rely on physicochemical indicators, such as peroxide value (PV) and the thiobarbituric acid reactive substances (TBARS) assay; although widely used, these tests are time-consuming and require hazardous reagents [7,8]. As a complementary strategy, volatile profiles correlate strongly with indices including the p-anisidine value (*p*-AnV) and total polar compounds (TPC) [9]. The *p*-AnV indexes secondary oxidation products (e.g., aldehydes and certain ketones), whereas TPC measures the aggregate level of polar oxidation species—such as oxidized triglyceride monomers, dimers, higher polymers, and epoxy fatty acids [8]. These aldehydes and epoxy fatty acids are electrophilic molecules and have been associated with disruptions in cellular lipid metabolism [10]. Accordingly, volatile compounds generated during oil peroxidation can serve as proxy indicators of other toxic oxidation products and, by extension, as sentinel markers of the biosafety of oxidized oils. Methodologically, headspace solid-phase microextraction coupled with gas chromatography–mass spectrometry (HS-SPME–GC–MS), when combined with PLSR, leverages volatile fingerprints to predict oxidation parameters in walnut oil and has shown robust performance for tracking quality changes [11]. Beyond their role as markers of lipid oxidation, several studies have highlighted the biological toxicity of lipid-derived volatile and semi-volatile compounds. Reactive aldehydes derived from ω-6 polyunsaturated fatty acids, including electrophilic lipid peroxidation products such as trans,trans-2,4-decadienal and related volatile species, can induce oxidative stress, activate pro-inflammatory signaling pathways, and disrupt cellular homeostasis in both in vitro and in vivo models [12,13]. Taken together, these findings indicate that volatile oxidation products carry information not only about oil quality but also about the potential biological hazards of oxidized lipids.

However, most chemometric applications to date have modeled physicochemical oxidation indices—such as PV, TBARS, p-AnV, or TPC—as response variables, whereas biological endpoints are rarely incorporated into predictive models [14]. Consequently, studies that directly link comprehensive volatile fingerprints of oxidized edible oils to cell-based inflammatory responses remain scarce [15]. Integrating multiple classes of oxidation markers with multivariate analysis is therefore essential for accurately assessing the degree of oxidation and predicting health-relevant bioactivity, such as pro-inflammatory potential.

Here, we used MLO as a chemical model and induced oxidation under accelerated storage (60 °C, 0–96 h) and high-temperature cooking (180 °C, 0–12 h) conditions. Free radicals, volatile compounds, and lipophilic aldehydes were profiled by electron spin resonance (ESR), HS-SPME-GC-MS, and ultra-performance liquid chromatography-tandem mass spectrometry (UPLC-MS/MS), respectively. Pro-inflammatory responses in RAW264.7 murine macrophages were quantified by RT-qPCR and ELISA (TNF-α, IL-1β, COX-2), along with oxidative stress indices, including reactive oxygen species (ROS), nitric oxide (NO), MDA and total superoxide dismutase (SOD) activity. We then trained and externally validated PLSR models to predict macrophage inflammatory responses from volatile fingerprints. Therefore, the objective of this study was to evaluate whether the volatile oxidation profile of MLO can be used to predict its pro-inflammatory capacity during processing and storage.

## 2. Materials and Methods

### 2.1. Materials and Reagents

MLO, ≥98% and 5,5-dimethyl-1-pyrroline-N-oxide (DMPO, 99%) were obtained from Sigma (USA). Valeraldehyde (98%), hexanal (≥99%, GC), (2E)-2-octenal (95%), (2E,4E)-deca-2,4-dienal (>90%, GC), aldehyde standards (≥95%), 1,1,3,3-tetramethoxypropane (TMP, 97%), (2E)-4-hydroxy-2-nonenal (≥97%), cyclohexanone (≥99.7%), and 2,4-dinitrophenylhydrazine (DNPH) were sourced from Macklin (China). The RAW264.7 murine macrophage cell line was provided by the Shanghai Institute of Cell Biology, Chinese Academy of Sciences. Methyl thiazolyl tetrazolium (MTT) was purchased from Beyotime Biotechnology (Shanghai). Sodium lauryl sulfate (SDS) and Tween 20 (99% biochemical grade) were acquired from Macklin Biochemical Technology (Shanghai, China). Polymerase chain reaction (PCR) primers were synthesized by Sangon Biotech (Shanghai, China). The RT-PCR kit and TransZol Up lysis reagent were obtained from TransGen Biotech (Beijing, China). The SYBR Green Realtime PCR Master Mix kit was provided by Toyobo (Shanghai, China). The ELISA kits were supplied by Jiangsu Enzyme Immunoassay Co., Ltd. (Wuxi, China). The detection kit for detecting MDA, ROS, NO and SOD activity was provided by Bayes Biotechnology (Shanghai, China) Company. All other chemicals and reagents were of analytical grade.

### 2.2. Preparation of Oxidized MLO Samples Under Different Temperatures

The accelerated oxidation of MLO was performed following the American Oil Chemists’ Society Official Method Cg 5-97 (AOCS, 1997) [16]. For oxidation at 60 °C [6], samples were prepared by weighing 1.00 ± 0.01 g of fresh MLO into an open amber vial and incubating it in a drying oven (Tianjin Zhonghuan, Tianjin, China) maintained at 60 °C under static air. The samples were agitated every 6 h. Aliquots were collected at predetermined time intervals of 0, 12, 24, 48, 72, and 96 h. Upon collection, each vial was immediately purged with nitrogen gas, sealed, and stored in the dark at −18 °C for subsequent analysis.

For high-temperature oxidation at 180 °C [9], MLO samples were prepared by weighing 1.00 ± 0.01 g of fresh MLO into an open brown ampoule. The ampoule was then immersed in a thermostatted oil bath (Shanghai Yiheng, Shanghai, China) containing 3 L of dimethyl silicone oil and maintained at 180 °C. During heating, the ampoules were kept open to ambient air, and no additional oxygen was supplied. Heating was continued for 12 h, with aliquots withdrawn at 0, 1, 2, 4, 8, and 12 h. Immediately after collection, each sample was cooled in an ice-water bath for 10 min, transferred to a 2 mL amber vial, purged with nitrogen, sealed, and stored in the dark at –18 °C pending analysis.

### 2.3. Free Radical Analysis

The composition and content of free radicals in MLO oxidized at 60 °C and 180 °C were detected using ESR [17]. Prepare DMPO toluene solution as a spin trap in the dark, and use it immediately. For MLO samples oxidized at 60 °C in a drying oven, take 100 μL at 0, 12, 24, 48, 72, and 96 h, add DMPO (final concentration 50 mM), transfer to an ESR tube, shake, load, and record spectrum. The pretreatment of MLO samples oxidized at different temperatures is the same before detection. ESR parameters: 3360 G center field, 20 mW microwave power, 100 G scan width, 1 min scan time, 1024 points of resolution, 100 kHz modulation frequency, 1.0 G modulation amplitude, 1.28 ms conversion time, 20.48 ms time constant, room temperature detecting temperature, and dark analysis.

### 2.4. Analysis of Volatile Compounds

After oxidizing the MLO at 60 °C and 180 °C, the volatile oxidation products were analyzed using HS-SPME-GC-MS, with cyclohexanone as the internal standard [18,19]. Weigh 0.2 ± 0.01 g of the MLO sample into a 20 mL headspace vial, and add cyclohexanone (500 μg/mL in methanol) as the internal standard. After 15 min of equilibration at 60 °C, use a divinylbenzene/carboxen/polydimethylsiloxane (DVB/CAR/PDMS) fiber to extract the volatiles for 40 min at 60 °C. Desorb the SPME fiber in splitless mode for five min at 250 °C in a GC injector. Take three samples and obtain three separate SPME extracts. A GC-MS/MS system (Agilent, Santa Clara, CA, USA) equipped with a DB-WAX column (30 m × 0.25 mm, 0.25 μm) was used for the analysis. A polar DB-WAX column was selected because it is widely used for the separation of aldehydes, esters, and other oxygenated volatiles in oxidized oils and provides good resolution for linoleate-derived oxidation products [20].

Helium (99.99%) was used as the carrier gas at a flow rate of 1 mL/min. The program temperature was set to 40 °C for 3 min, then ramped up to 125 °C at 8 °C/min, to 165 °C at 3 °C/min, and to 230 °C at 10 °C/min for 2 min. The mass spectrometer (MS) specifications included an electron energy of 70 eV, a scan range of 50–550 *m*/*z*, an electron multiplier voltage of 1500 V, a temperature of 230 °C for the ion source, 250 °C for the transfer line, and 150 °C for the quadrupole. Chromatographic peak regions were compared to those of cyclohexanone to calculate relative concentrations. Linear retention indices (RIs) were calculated using a C8–C40 n-alkane series injected under the same conditions, and experimental RIs and mass spectra were compared with the NIST 14 and Wiley 8.0 libraries to support chromatographic selectivity and tentative identification of volatile compounds.

Quantitative analysis: Known amounts of the internal standard (cyclohexanone) were added during the analysis. By comparing the peak area of each volatile compound with that of the internal standard, the proportional content of each compound was determined.

$$C_{i}=\frac{S_{i}}{S_{a}}C_{a}$$

In this case, the letters *C_i_*, *S_i_*, *C_a_*, and *S_a_* stand for the concentration of the unknown molecule, the internal standard, and the peak area of the internal standard, respectively.

### 2.5. Analysis of Lipophilic Aldehydes

The quantification of lipophilic aldehyde compounds was conducted following an established methodology [21], which involves the derivatization of lipophilic aldehydes in MLO. The derivatization procedure is as follows: Prepare a 2.5 mM DNPH solution with methanol-concentrated hydrochloric acid (9:1, *v*/*v*). Weigh 0.2 ± 0.01 g of MLO sample under different oxidation conditions and react with 1 mL of freshly prepared DNPH reagent. After the reaction, add 2 mL of methanol-water (75:25, *v*/*v*), vortex to extract the derivatives of DNPH and aldehydes, centrifuge at 5000× *g* for 5 min, separate the layers, and collect the methanol layer. Repeat the process once. Combine the methanol layers, add 3.5 mL of water, extract the DNPH derivatives with 2 mL of CH_2_Cl_2_, centrifuge at 5000× *g* for 5 min, separate the layers, and collect the CH_2_Cl_2_ layer. Repeat this procedure once more. Combine the CH_2_Cl_2_ layers, dry with nitrogen, redissolve in 2 mL of methanol, and vortex for 2 min to ensure complete dissolution. Derivatize standard solutions simultaneously with MLO samples.

The treated samples were analyzed using UPLC-MS/MS. Preliminary qualitative analysis was performed by reviewing the literature, while quantitative analysis was conducted using the external standard method. This method utilized five common aldehyde compounds from linoleic acid oxidation as standards: valeraldehyde, hexanal, (2E)-2-octenal, (2E,4E)-2,4-nonadienal, and (2E)-4-hydroxy-2-nonenal. The DNPH derivatization–extraction protocol was adopted from an established method [22], which has been validated for the quantification of DNPH derivatives of lipid oxidation aldehydes. Chromatographic conditions were as follows: Waters BEH C18 column (100 mm × 2.1 mm, 1.7 μm); mobile phase A: ultrapure water, mobile phase B: methanol; flow rate: 0.30 mL/min; injection volume: 5 μL; gradient elution: hold at 100% B for 2 min, 7.5–10 min (100–70% B), 0–2.5 min (70% B), 2.5–3.5 min (70–80% B), 3.5–4.5 min (80–90% B), and 4.5–5.5 min (90–100% B). The mass spectrometry settings were as follows: ESI ion source in negative ion mode, 3.0 kV for the capillary, 150 °C for the source, 550 °C for the desolvation gas, cone gas flow rate: 150 L/h, cone voltage: 15 V, collision energy: 20 eV, and flow rate: 950 L/h.

### 2.6. Preparation of Oxidized MLO Emulsions

The solubility of MLO in cell culture medium is relatively low. To achieve mutual solubility between MLO and the culture medium, the commonly used emulsifier Tween 20 (TW20) was employed in this study [23]. A Zetasizer Nano ZS90 laser particle analyzer (Bettersize, Dandong, China) was used to assess the particle size and zeta potential of the MLO-TW20 emulsion. Emulsions were prepared with varying concentrations of TW20 (0, 0.5%, 1%, 2%, 5%, 10%, 20%, 30%, and 40%, m/m) and diluted 100-fold with ultrapure water. The resulting mixtures were uniformly mixed, and 1 mL of the diluted sample was transferred into cuvettes and electrophoretic cells for measurement. All measurements were performed at 25 °C with a 10 s equilibration time. The parameters for the dispersed phase, dispersion medium, and measurement mode were specifically configured according to the physicochemical properties of MLO.

### 2.7. Cell Culture

RAW264.7 mouse macrophages were cultured in DMEM high-glucose medium with 10% FBS and 1% penicillin/streptomycin at 37 °C in a 5% CO_2_ incubator. When cells reached 80–90% confluence, they were passaged for subsequent experiments.

### 2.8. Determination of Cell Viability

The MTT assay was employed to assess the viability of RAW264.7 cells treated with oxidized MLO products. RAW264.7 cells were seeded in 96-well plates at a density of 2 × 10^4^ cells per well. The cells were exposed to oxidized MLO emulsions ranging from 0 to 1000 μg/mL for durations of 8, 12, and 24 h. After adding 10 μL of MTT solution to each well, the wells were incubated for 4 h at 37 °C in a 5% CO_2_ atmosphere. Following the dissolution of the resultant formazan crystals in 100 μL of SDS solution, absorbance was measured at 570 nm using a multimode microplate reader.

### 2.9. Measurement of Inflammatory Factors at Gene and Protein Expression Levels

The relative expression levels of inflammatory factors were quantitatively measured by RT-PCR. Cells in the logarithmic growth phase were seeded in 6 cm cell culture dishes at a density of 2.4 × 10^6^ cells. After reaching the logarithmic growth phase, the cells were treated with different concentrations of oxidized MLO for 8 and 12 h to preliminarily screen the optimal treatment concentration and time for constructing a cellular inflammatory model based on the gene expression levels of inflammation-related factors. Subsequently, the cells were treated with MLO obtained under different oxidation conditions, with cells without any treatment serving as the blank control group.

After the acquisition of RAW264.7 cells, total RNA was extracted and purified according to the instructions provided with the Gen Trans RNA Extraction Kit. The RNA concentration was then quantified using a Q5000 UV-visible spectrophotometer. Reverse transcription of RNA was performed using a reverse transcription kit. The reaction was carried out in a PCR amplifier at 37 °C for 15 min, 50 °C for 5 min, and 98 °C for 5 min. The resulting cDNA was stored at −20 °C for subsequent use. The primer sequences are shown in Table 1. Using β-actin as the internal reference, the 2^(−ΔΔCt)^ method was used to determine the relative gene expression levels [24].

Determination of protein secretion: After collecting the cell supernatant, the protein secretion level of pro-inflammatory related factors in cells was detected according to the ELISA kit instructions.

### 2.10. Determination of Oxidative Stress-Related Indicators

In accordance with Section 2.7, RAW264.7 cells were seeded in 96-well plates or 6 cm dishes and cultured until they reached >80% confluence. The cells were treated with MLO produced under various oxidation conditions for 8 h at a concentration of 200 μg/mL, following a 24 h incubation period. Cells that received no treatment served as the blank control group. After collecting the cells, the ROS, MDA, and Total SOD Activity Assay Kits were used to measure ROS levels, MDA levels, and SOD enzyme activity in the samples. After collecting the cell supernatant, the Total NO Assay Kit was used according to the manufacturer’s instructions to measure the NO level in the samples.

### 2.11. Statistical Analysis

All experiments were performed in triplicate, and the data are expressed as the mean ± standard deviation. The data obtained in this study were initially organized using Microsoft Excel and then analyzed statistically using SPSS Statistics 26 software. Analysis of variance (ANOVA) and Duncan’s multiple range test were used to compare the significance of differences between data sets, with *p* < 0.05 indicating a significant difference. Orthogonal partial least squares discriminant analysis (OPLS-DA) was performed in SIMCA 14.1. PLSR models were calibrated using a training set and evaluated by leave-one-out cross-validation and external validation on an independent test set. All statistical analyses were carried out using the Unscrambler X 10.4, and graphs were generated using Origin 2024.

## 3. Results and Analysis

### 3.1. Changes in Free Radicals During MLO Oxidation

**Figure 1:**
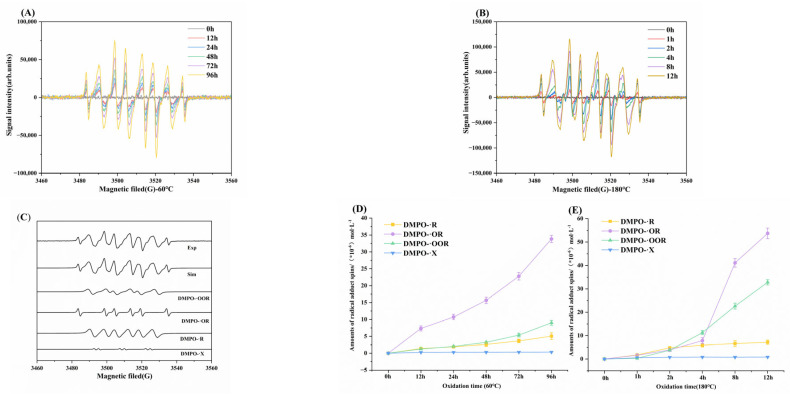
Changes in free radicals in MLO during the oxidation process. (**A**) −60 °C ESR plots; (**B**) −180 °C ESR plots; (**C**) Free radical fitting plots (Exp. for experimental plots, Sim. for fitting plots); (**D**) −60 °C Specific free radical concentration; (**E**) −180 °C Specific free radical concentration.

The degradation of MLO is primarily driven by a free radical chain reaction during lipid oxidation [17]. Due to the high reactivity and short-lived nature of the resulting radicals, direct detection via ESR spectroscopy is challenging. Therefore, spin-trapping agents—typically containing nitroso or nitrone functional groups—are employed to stabilize these species by forming relatively persistent nitroxide spin adducts. Among these, DMPO is widely preferred for its ability to generate stable adducts with both carbon-centered and oxygen-centered radicals, making it a key reagent for elucidating radical-mediated reaction pathways [25]. In this study, the temporal evolution of free radicals during thermal oxidation of MLO was analyzed using ESR spectroscopy combined with DMPO spin-trapping. As illustrated in Figure 1A,B, the ESR spectra of MLO oxidized at 60 °C and 180 °C displayed a marked increase in signal intensity compared to the unoxidized control (stable baseline). Furthermore, signal intensity grew progressively with oxidation time, consistent with Zhang’s findings [26], confirming that radical accumulation is positively correlated with the degree of oxidation [27].

The center of the free radical spectrum, the g-value, is essential for determining the type of free radical [28]. Using Xenon software for fitting, similar results were obtained for the free radical signals of MLO trapped by DMPO under both oxidation conditions. Figure 1C displays the fitted spectrum of radical signals after 12 h of oxidation at 180 °C. A comparison between the experimental and simulated spectra confirms excellent fitting accuracy, revealing that the DMPO spectrum is composed of four distinct radical species [29]. The spin adducts are identified as follows: alkyl radicals (DMPO/R: g = 2.00592, aN = 14.98 G, aH = 20.68 G), alkoxy radicals (DMPO/OR: g = 2.00589, aN = 13.89 G, aH = 7.98 G), peroxy radicals (DMPO/OOR: g = 2.00586, aN = 13.87 G, aH = 10.16 G), and secondary free radical adducts formed after the spin–trap captures the free radical (DMPO/X: g = 2.00602, aN = 14.82 G).

Figure 1D,E illustrate changes in the concentrations of various free radicals in MLO as a function of oxidation time. Free radicals were detectable in MLO at the start of oxidation, and their concentrations continued to increase, indicating that the formation rate of spin adducts exceeded the decay rate [30]. At 60 °C (Figure 1D), over 96 h of oxidation, the predominant radical was RO, with its concentration 6.69 times that of ROO· at the end. At 180 °C (Figure 1E), over 12 h of oxidation, the predominant radicals were RO· and ROO. After 4 h, RO increased sharply, whereas ROO· increased approximately linearly; at the end, the RO· concentration was 1.64 times that of ROO·. R· and the unknown radical X· remained at low levels. At 180 °C, R· was initially dominant, but after 4 h it was surpassed by ROO· and RO. In contrast, at 60 °C, RO· remained consistently higher than the otherthree radicals, indicating temperature-dependent oxidation mechanisms [31].

### 3.2. Changes in Volatile Compounds During MLO Oxidation

Volatile compounds are generated during MLO oxidation. This study employed HS-SPME-GC-MS to analyze the evolution of volatile oxidation products of MLO at 60 °C and 180 °C [32], with the results presented in Table 2 and Table 3.

**Figure 2:**
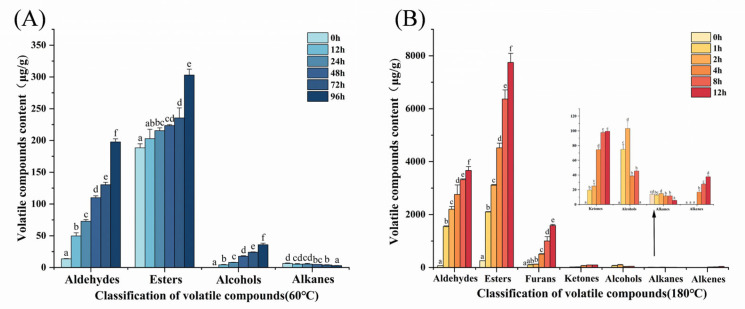
Changes in volatile oxidation products during the oxidation process of MLO. (**A**) −60 °C; (**B**) −180 °C.

At 60 °C, 21 compounds were identified, including 7 aldehydes, 2 alcohols, 9 esters, and 3 alkanes. Unoxidized MLO exhibited minimal volatile compounds, indicating low initial oxidation. Dynamic analysis (Figure 2A) revealed aldehydes and esters as the predominant products, whose relative abundances increased significantly over time (*p* < 0.05), peaking at 96 h. Notably, hexanal, heptenal, and (2E,4E)-deca-2,4-dienal showed marked increases, reaching 46.17 ± 3.10 μg/g, 89.00 ± 3.67 μg/g, and 30.17 ± 3.80 μg/g, respectively, at the end of oxidation. Heptenal and (2E,4E)-deca-2,4-dienal were detected even in unoxidized samples, highlighting their propensity to form. Among the detected esters, methyl octylate was the most abundant (228.89 ± 13.60 μg/g) at the end of oxidation (Table 2). At 180 °C, a total of 33 compounds were identified—7 aldehydes, 2 alcohols, 3 ketones, 14 esters, 3 furans, 2 alkanes, and 2 alkenes—indicating greater product complexity than at 60 °C, particularly for alcohols, ketones, and furans. This pattern suggests that oxidation and cracking of long-chain compounds proceed more readily at 180 °C, yielding smaller molecules. As shown in Figure 2B, the abundances of aldehydes and esters changed significantly over time, with aldehydes, ketones, furans, and esters reaching their maxima at 12 h. Notably, heptenal, (2E)-2-octenal, and (2E,4E)-deca-2,4-dienal showed the most significant changes, reaching 568.92 ± 10.64 μg/g, 511.40 ± 23.08 μg/g, and 2207.03 ± 112.83 μg/g, respectively, at the end of oxidation. Esters were more diverse at high temperature; however, methyl laurate was detected only in unoxidized MLO (Table 3).

These results align with Sun et al.’s [32] report on the thermal oxidation behavior of plant oils, confirming a positive correlation between the content of volatile compounds and heating time. In particular, linoleic acid’s characteristic oxidation products (e.g., hexanal, (2E,4E)-deca-2,4-dienal) exhibited significant time-dependent accumulation during accelerated oxidation. Moreover, the greater complexity of products at 180 °C compared to 60 °C is consistent with Xu et al.’s [57] findings on the effects of temperature on oil oxidation pathways.

### 3.3. Changes in Lipophilic Aldehydes During MLO Oxidation

Analysis of free radicals and volatiles in MLO oxidation revealed that the degree of oxidation increased with oxidation time under both conditions, with most volatile compounds exhibiting upward trends, suggesting a potential positive correlation between lipophilic aldehyde types and the degree of oxidation. To test this, we derivatized MLO samples oxidized at 180 °C for 12 h and used UPLC-MS/MS for qualitative analysis, identifying 15 lipophilic aldehydes (Table 4). Carbon chain length significantly affected retention times [58].

An external standard method was used to quantify five typical aldehydes (hexanal, heptenal) from linoleic acid oxidation. Their standard curves exhibited R^2^ > 0.99 (Table S1), indicating good linearity and suitability for aldehyde content analysis during MLO oxidation. It should be noted that a full spike–recovery assessment of DNPH-derivatized aldehydes in the MLO matrix was not performed in this study; therefore, the reported aldehyde concentrations should be interpreted as semi-quantitative and are most appropriate for comparing oxidation time and conditions rather than for absolute quantification.

**Figure 3:**
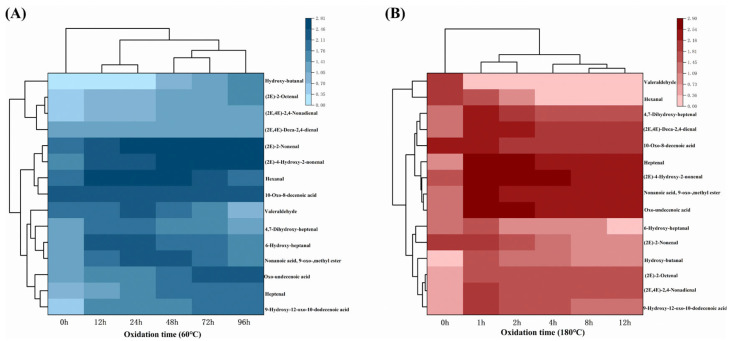
Changes in lipophilic aldehyde compounds during the oxidation process of MLO. (**A**) −60 °C; (**B**) −180 °C.

Figure 3 illustrates significant differences in lipophilic aldehyde content under various oxidation conditions. Fifteen carbonyl compounds were detected in fresh, unoxidized MLO (Figure 3A), but at low levels, likely due to minimal oxidation of ω-6 fatty acids during processing, transport, or storage [12]. At 60 °C, aldehyde content (9-hydroxy-12-oxo-10-dodecenoic acid, (2E,4E)-deca-2,4-dienal) increased with oxidation time (0–96 h), peaking at 96 h. This aligns with the self-catalytic chain reaction mechanism of lipid oxidation: initial radical attack on fatty acids forms hydroperoxides, which further degrade into aldehydes [30]. At 180 °C (Figure 3B), except for hexanal and valeraldehyde, the other 13 lipophilic aldehydes spiked to maximum levels at 1 h and then declined with oxidation time. This pattern suggests that these aldehydes are transient intermediates at high temperature and are further transformed (e.g., by volatilization and oxidation to other products), resulting in decreased measured concentrations despite ongoing oxidation. This is consistent with Yao et al.’s [30] findings that prolonged heating at high temperatures alters fatty acid oxidation sites and related aldehyde types.

### 3.4. Effects of Oxidized MLO on Inflammatory Factor Gene Expression

To assess the impact of oxidized MLO on macrophage inflammation, we first determined its safe concentration range via cytotoxicity tests. The results showed that cells maintained over 85% viability when treated with oxidized MLO (from both oxidation conditions) at 10–200 μg/mL for 8 or 12 h. Additionally, 52.4 μg/mL Tween 20 (the highest emulsifier concentration used) did not affect cell viability. Based on these results, we selected concentrations of 10, 50, 100, and 200 μg/mL and incubation times of 8 and 12 h to assess the expression of inflammatory factors TNF-α, IL-1β, and COX-2. After treating cells with oxidized MLO at different concentrations for 8 h, all three inflammatory factors increased compared to the blank control, with a significant elevation observed at 200 μg/mL (*p* < 0.05). Notably, the 8 h treatment group exhibited higher inflammatory factor expression levels than the 12 h group at the same concentration. Therefore, for subsequent experiments under both oxidation conditions, we chose to incubate cells with 200 μg/mL oxidized MLO for 8 h (Supplementary Figures S1 and S2).

**Figure 4:**
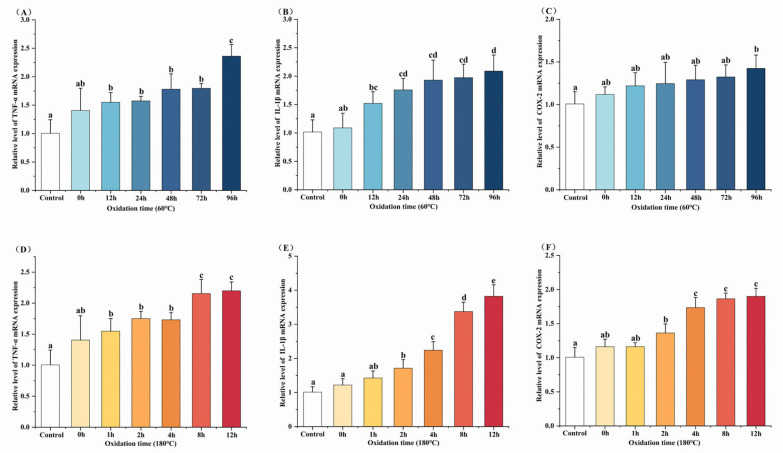
Effect of oxidized MLO on inflammatory gene expression in RAW264.7 cells. Different letters indicated significant differences (*p* < 0.05). (**A**–**C**), 60 °C; (**D**–**F**), 180 °C.

We evaluated the effects of oxidized MLO under different conditions on the mRNA expression of pro-inflammatory factors TNF-α, IL-1β, and COX-2 in RAW264.7 cells. RT-qPCR results (Figure 4) showed that after cells were stimulated with MLO oxidized at 60 °C for 96 h, mRNA levels of all three factors increased significantly compared to the blank control (*p* < 0.05). Fresh, unoxidized MLO had no such effect. Similarly, MLO oxidized at 180 °C for 12 h also caused significant increases in mRNA levels of the three factors (*p* < 0.05). These results indicate that oxidized MLO induces pro-inflammatory factor gene expression, which is dependent on its concentration, treatment time, cytokine type, and oxidation time.

### 3.5. Effects of Oxidized MLO on Inflammatory Factor Protein Expression

**Figure 5:**
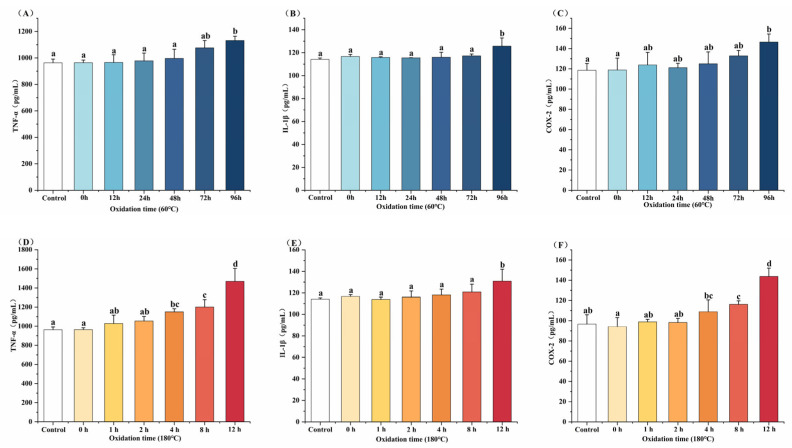
Effect of oxidized MLO on protein expression of inflammatory factors in RAW264.7 cells. Different letters indicated significant differences (*p* < 0.05). (**A**–**C**), 60 °C; (**D**–**F**), 180 °C.

ELISA was used to assess the impact of oxidized MLO under different conditions on the protein expression of pro-inflammatory factors TNF-α, IL-1β, and COX-2 in RAW264.7 cells (Figure 5). As oxidation time increased, protein levels of these factors rose in cells treated with oxidized MLO under both conditions. The most significant increases were observed with MLO oxidized at 60 °C for 96 h and 180 °C for 12 h compared to the blank control (*p* < 0.05), consistent with the mRNA expression trends. These findings suggest that as oxidation time increases, the pro-inflammatory potential of oxidized MLO rises under both conditions. This could be due to the accumulation of toxic, complex metabolic products in oxidized oils, which induce inflammatory responses in cells [13]. Protein levels were assessed by ELISA in parallel with mRNA measurements; however, additional confirmation by Western blotting was not performed and should be pursued in future work.

### 3.6. Effects of Oxidized MLO on Oxidative Stress—Related Parameters in RAW264.7 Cells

**Figure 6:**
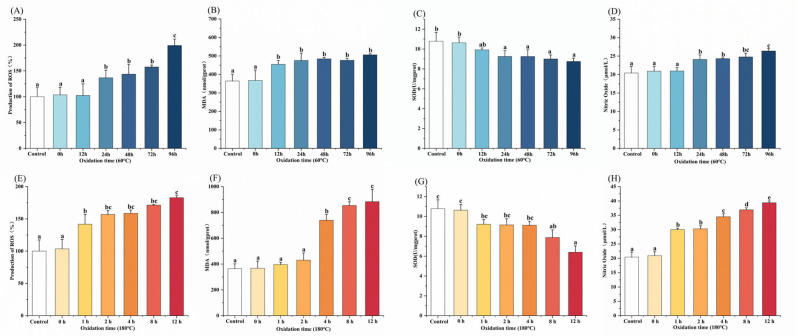
Effect of oxidized MLO on oxidative stress in RAW264.7 cells. Different letters indicated significant differences (*p* < 0.05). (**A**–**D**), 60 °C; (**E**–**H**), 180 °C.

Oxidative stress, caused by an imbalance between oxidants and antioxidants, can damage cellular structures and contribute to chronic inflammatory diseases such as cardiovascular disease, diabetes, and cancer [13,59]. This study comprehensively evaluated the effects of oxidized MLO on oxidative stress-related parameters in RAW264.7 cells. ROS, a key mediator of oxidative stress [60], were quantitatively assessed using the DCFH-DA fluorescence probe method. As shown in Figure 6A,E, compared to the blank control (CK) group, oxidized MLO significantly elevated intracellular ROS levels in RAW264.7 cells, with ROS accumulation showing dependence on oxidation time (*p* < 0.05). No significant difference was found between the unoxidized MLO-treated group and the CK group (*p* > 0.05). MDA, a product of lipid peroxidation, reflects the degree of membrane lipid oxidation under oxidative stress [61]. The TBARS colorimetric method was used to measure intracellular MDA content. As shown in Figure 6B,F, compared to the CK group, MLO oxidized for the longest duration under both conditions significantly increased intracellular MDA production (*p* < 0.05). SOD, a core enzyme in the antioxidant defense system [62], has its activity regulated by oxidized MLO, with significant differences observed. As shown in Figure 6C,G, compared to the CK group, MLO oxidized for different times under both conditions had varying inhibitory effects on SOD activity, which were dependent on oxidation time (*p* < 0.05). NO, a key mediator in inflammation, was measured by the Griess method as an indicator of the inflammatory response [63]. As shown in Figure 6D,H, compared to the CK group, most oxidized MLO samples significantly increased NO release (*p* < 0.05), with the inducing effect intensifying with longer oxidation times. 

### 3.7. OPLS-DA Analysis of MLO Oxidation Products

During lipid thermal processing, the oxidation duration plays a crucial role in controlling the dynamics of volatile chemical production [64]. Using OPLS-DA, the relationship between volatile chemicals (dependent variables) and oxidation duration (independent variable) was modeled to identify differences among samples oxidized under various conditions.

**Figure 7:**
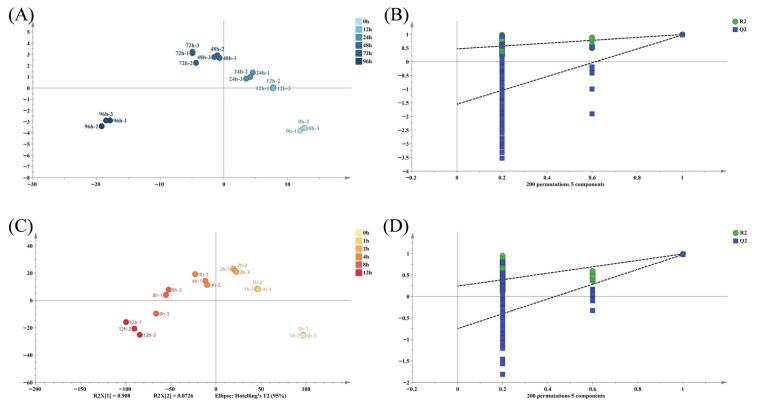
OPLS-DA analysis of MLO oxidation products. (**A**) −60 °C Orthogonal partial least squares discrimination results; (**B**) −60 °C 200 permutation test results; (**C**) −180 °C Orthogonal partial least squares discrimination results; (**D**) −180 °C 200 permutation test results.

At 60 °C, the OPLS-DA model demonstrated excellent fit (R^2^y = 0.966) and predictability (Q^2^ = 0.81) (Figure 7B). All data points fell within the 95% confidence interval, with clear clustering and no outliers (Figure 7A). Similarly, at 180 °C, the model parameters (R^2^y = 0.931, Q^2^ = 0.814) (Figure 7D) and sample distribution (Figure 7C) confirmed good classification. In both cases, oxidized samples moved away from unoxidized ones along the first principal component with increasing oxidation time, indicating that the OPLS-DA model could classify MLO samples based on oxidation time. This aligns with lipid oxidation theory, where oxidation time regulates the progression of free radical chain reactions, driving the conversion of primary products (e.g., hydroperoxides) to secondary ones (aldehydes, ketones, etc.) [32]. Despite higher reaction kinetics at 180 °C [31], the similar classification ability of the model at both temperatures indicates a universal association between volatile compound formation and oxidation time.

### 3.8. Correlation Analysis of MLO Oxidation Products

**Figure 8:**
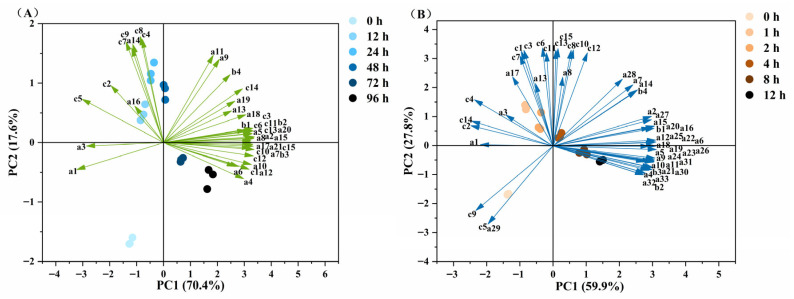
Correlation analysis of MLO oxidation products (a. Volatile Compounds; b. free radical; c. lipophilic aldehyde compounds). (**A**) −60 °C; (**B**) −180 °C.

To understand the interactions between free radicals, volatile compounds, and lipophilic aldehydes during MLO oxidation, principal component analysis (PCA) [65] was used to reduce the dimensionality of the data set. Figure 8A shows the biplot of the first two principal components (PC1 and PC2) for MLO oxidized at 60 °C, accounting for 88% of the variance. PC1 was primarily related to volatile compounds and PC2 to lipophilic aldehydes based on variable loadings. The biplot revealed clear associations between sample positions and oxidation products, as well as among the three classes of variables. Decane and undecane were associated with fresh MLO samples (along negative PC1), whereas alkyl radicals, alkoxy radicals, (2E)-4-hydroxy-2-nonenal, (2E)-2-octenal, and (2E,4E)-deca-2,4-dienal were aligned with late-stage samples (positive PC1), indicating that these compounds can reflect quality changes in oxidized MLO. Figure 8B shows the biplot of PC1 and PC2 for MLO oxidized at 180 °C, accounting for 87.7% of the variance. Consistently, oxidation characteristics were reflected by free radicals, hexanal, 2-butylfuran, 2-amylfuran, methyl heptanoate, (2E)-2-octenal, (2E,4E)-deca-2,4-dienal, methyl 9-oxononanoate, and (2E)-4-hydroxy-2-nonenal. Aldehydes such as (2E)-4-hydroxy-2-nonenal and (2E,4E)-deca-2,4-dienal are known to play roles in inflammation-related chronic metabolic diseases [59,66]. Thus, analyzing volatile compound formation during lipid oxidation can indicate oil quality changes and is crucial for understanding food-safety risks of oxidized oils.

### 3.9. Analysis of Pro-Inflammatory Predictive Models for Oxidized MLO

This study demonstrates a substantial association between volatile chemicals and other oxidation products in oxidized oils, highlighting their potential as effective markers of oil quality deterioration, based on the compositional changes observed during the MLO oxidation process. HS-SPME–GC–MS provided efficient extraction and enrichment of volatile components [67]. whereas RT-qPCR offered high sensitivity and resolution, enabling precise detection of subtle variations in inflammatory gene expression. To quantify the relationship between oxidation products and pro-inflammatory effects, a PLSR model was developed using data collected at six oxidation time points [68]. In this model, the contents of volatile compounds were set as the independent variables (X-matrix), and the gene expression levels of inflammatory factors served as the dependent variables (Y-matrix). The samples were divided into training and test sets using a sample set partitioning algorithm, and the model’s robustness was assessed via leave-one-out cross-validation [69].

The discrepancy between expected and actual values is measured by the root mean square error (RMSE); a model with a smaller RMSE is considered more accurate [70]. R^2^ quantifies the fit of the linear regression model to the observed data. Models can be classified as follows: low correlation (0.26 < R^2^ < 0.49), high precision (R^2^ > 0.90), adequate for approximate sample prediction (0.65 < R^2^ < 0.81), good correlation (0.82 < R^2^ < 0.90), and the ability to distinguish between low and high sample values (0.50 < R^2^ < 0.64) [71]. A higher relative percent difference (RPD) indicates better quantitative prediction ability, with an RPD > 2 suggesting reliable model predictions [72].

**Figure 9:**
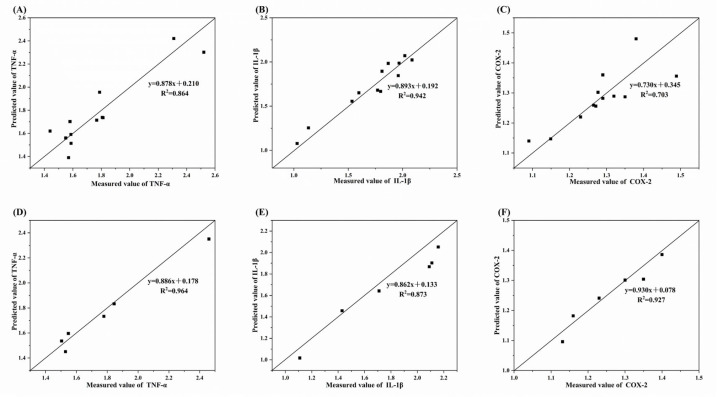
Internal and external verification results of the predicted and measured values of inflammatory genes under oxidation conditions at 60 °C. (**A**–**C**), Internal verification result; (**D**–**F**), External verification result.

The PLSR model under 60 °C oxidation conditions demonstrated strong predictive performance through both internal and external validation. Internal calibration and cross-validation yielded low root mean square errors of cross-validation (RMSECV: 0.125 for TNF-α, 0.085 for IL-1β, 0.059 for COX-2) and high cross-validated coefficients of determination (R^2^CV: 0.864, 0.942, 0.703, respectively; see Table 5 and Figure 9A–C). The high RPD values (2.599, 3.966, 1.743) further confirmed the model’s reliability. External validation reinforced these results, showing strong linear relationships between predicted and measured values for all three inflammatory factors, with high R^2^P values (0.964, 0.873, 0.927; Figure 9D–F) and minimal prediction bias (RMSEP: 0.063, 0.140, 0.026, all near zero).

**Figure 10:**
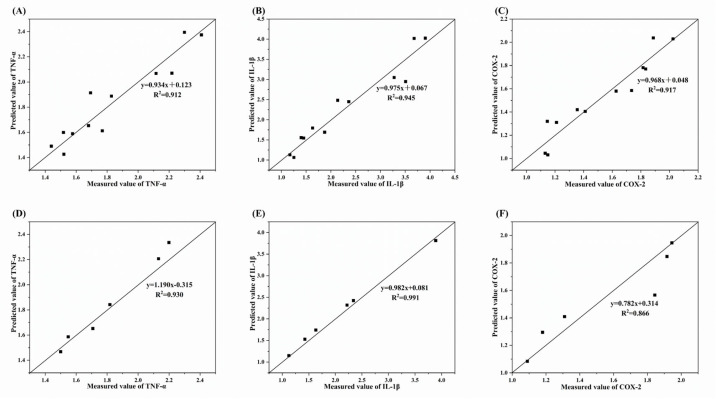
Internal and external verification results of the predicted and measured values of inflammatory genes under oxidation conditions at 180 °C. (**A**–**C**), Internal verification result; (**D**–**F**), External verification result.

Under 180 °C oxidation, the PLSR results (Table 6) showed RMSECV values of 0.104, 0.250, and 0.099, and R^2^CV values of 0.912 (Figure 10A), 0.945 (Figure 10B), and 0.917 (Figure 10C). The RPD values of 3.230, 4.079, and 3.330, as indicated by the strong R^2^ values, demonstrate the accuracy and predictive capability of the regression model. External validation was performed using six samples not included in the modeling. Figure 10 compares measured and predicted values from cell experiments. For TNF-α, IL-1β, and COX-2, the R^2^P values were 0.930 (Figure 10D), 0.991 (Figure 10E), and 0.866 (Figure 10F), and the RMSE values were 0.070, 0.093, and 0.132, respectively. The PLSR model’s ability to predict the pro-inflammatory effects of oxidized MLO is demonstrated by these results, which also show that volatile molecules derived from lipid oxidation are indicative of the pro-inflammatory effects of oxidized oils.

## 4. Conclusions

In this study, chromatography–mass spectrometry, electron spin resonance, and chemometric analysis were combined to characterize the oxidation behavior and pro-inflammatory effects of MLO under storage-like (60 °C) and high-temperature (180 °C) conditions. With increasing oxidation time, free radical species, volatile compounds, and lipophilic aldehydes showed distinct temperature-dependent patterns, with most lipophilic aldehydes peaking at 96 h at 60 °C and at 1 h at 180 °C. Oxidized MLO markedly enhanced TNF-α, IL-1β, and COX-2 expression and increased ROS, NO, and MDA while decreasing SOD activity in RAW264.7 macrophages, indicating that higher oxidation levels are associated with stronger pro-inflammatory and pro-oxidant responses.

PLSR models built from volatile fingerprints quantitatively predicted these inflammatory endpoints, with R^2^ values above 0.85 for TNF-α, IL-1β, and COX-2. These results demonstrate that volatile oxidation products can serve as mechanism-linked markers of the pro-inflammatory capacity of oxidized MLO and lay a foundation for predicting inflammatory potency based on odor-related volatile signatures.

As the models were developed using MLO as a simplified lipid system, their direct application to complex edible oils and high-lipid foods will require additional calibration and validation across different matrices and oxidative conditions. At this stage, the modeling framework is best viewed as a rapid screening tool that can be adapted and recalibrated for specific oil or food categories, rather than as a universal industrial standard. Future studies should extend this approach to other lipid systems and complex food matrices and benchmark its performance against existing volatile-based chemometric models in order to assess its robustness and practical applicability.

## Figures and Tables

**Table 1:** Primer sequences.

Gene		Primer Sequences
β-actin	R	5′-TCATGAAGTGTGACGTTGACATCCGT-3′
	F	5′-CCTAGAAGCATTTGCGGTGCACGATG-3′
TNF-α	R	5′-ATGAGCACAGAAAGCATGATC-3′
	F	5′-TACAGGCTTGTCACTCGAATT-3′
IL-1β	R	5′-TGCAGAGTTCCCCAACTGGTACATC-3′
	F	5′-GTGCTGCCTAATGTCCCCTTGAATC-3′
COX-2	R	5′-AGAAGGAAATGGCTGCAGAA-3′
	F	5′-GCTCGGCTTCCAGTATTGAG-3′

**Table 2:** Volatile components and content changes in MLO under 60 °C oxidation conditions.

RT	Name	CAS	Actual RI	Theoretical RI	Content (μg/g)						References
					0 h	12 h	24 h	48 h	72 h	96 h	
8.706	Decane	124-18-5	1060	1000	1.73 ± 0.24	1.24 ± 0.15	0.93 ± 0.06	0.59 ± 0.06	0.46 ± 0.05	-	NIST Library
9.684	Hexanal	66-25-1	1110	1110	-	7.08 ± 0.12	12.90 ± 0.63	24.20 ± 1.30	28.82 ± 0.77	46.17 ± 3.10	[33]
10.714	Undecane	1120-21-4	1164	1100	4.75 ± 0.67	4.13 ± 0.80	4.18 ± 1.00	3.83 ± 0.07	3.22 ± 0.26	2.45 ± 0.22	NIST Library
11.767	3-hydroxy Stearic Acid Methyl ester	2420-36-2	1221	2239	-	-	-	-	1.75 ± 0.16	1.97 ± 0.42	NIST Library
14.113	Heptenal	18829-55-5	1354	1342	7.71 ± 0.35	22.41 ± 0.69	35.40 ± 1.24	55.74 ± 1.18	65.18 ± 0.84	89.00 ± 3.67	[34]
15.624	Methyl octylate	111-11-5	1437	1437	169.05 ± 10.01	170.91 ± 10.06	173.79 ± 2.39	176.10 ± 0.71	181.05 ± 14.93	228.89 ± 13.60	[35]
16.207	(2E)-2-Octenal	2548-87-0	1467	1467.3	-	3.86 ± 0.08	5.84 ± 0.03	8.51 ± 0.36	11.81 ± 0.47	23.62 ± 1.40	[36]
16.264	Trans-2-Octen-1-ol	18409-17-1	1470	1603	-	4.39 ± 0.57	8.03 ± 0.38	14.67 ± 0.86	19.99 ± 0.32	27.48 ± 1.81	[37]
17.809	Methyl nonanoate	1731-84-6	1544	1536	-	6.07 ± 0.35	6.51 ± 0.25	6.32 ± 0.07	6.56 ± 0.16	6.64 ± 0.16	[35]
18.353	3,5-Dimethylcyclohexanol	5441-52-1	1569	1809	-	-	-	3.02 ± 0.08	4.11 ± 0.32	8.40 ± 1.00	[38]
18.576	(2E)-2-Nonenal	18829-56-6	1579	1574	-	4.20 ± 0.16	4.16 ± 0.16	4.31 ± 0.06	3.63 ± 0.56	4.31 ± 0.73	[39]
20.338	Methyl Caprate	110-42-9	1654	1636	2.73 ± 0.72	2.70 ± 0.28	3.17 ± 0.40	2.74 ± 0.08	4.55 ± 0.18	4.53 ± 0.74	[35]
21.471	4-Decenoic acid methyl ester	1191-02-2	1700	1622	13.79 ± 4.21	16.54 ± 3.84	18.53 ± 2.87	18.88 ± 0.49	19.17 ± 0.83	22.39 ± 2.06	[40]
22.29	2-Butyloct-2-enal	13019-16-4	1731	1659	-	0.59 ± 0.19	0.79 ± 0.23	0.75 ± 0.05	-	-	[41]
22.461	(2E,4E)-2,4-Nonadienal	5910-87-2	1737	1738	-	-	1.40 ± 0.42	1.82 ± 0.13	2.67 ± 0.11	4.54 ± 0.54	[42]
23.108	Methyl undecanoate	1731-86-8	1761	1732	1.85 ± 0.88	2.24 ± 0.91	2.22 ± 0.10	2.98 ± 0.03	3.66 ± 0.58	-	[35]
25.511	(2E,4E)-Deca-2,4-dienal	25152-84-5	1849	1854	5.98 ± 0.19	11.55 ± 4.25	12.37 ± 1.02	14.61 ± 0.10	18.28 ± 0.97	30.17 ± 3.80	[33]
26.124	Methyl laurate	111-82-0	1871	1834	1.07 ± 0.06	1.44 ± 0.36	2.00 ± 0.19	2.39 ± 0.18	2.43 ± 0.12	2.99 ± 0.52	[35]
27.199	1,1-Dimethoxyoctane	10022-28-3	1908	1530	-	-	0.48 ± 0.07	0.44 ± 0.03	0.46 ± 0.02	0.55 ± 0.10	[33]
28.99	methyl 8-oxooctanoate	3884-92-2	1963	1334	-	-	0.88 ± 0.36	1.25 ± 0.03	1.40 ± 0.10	3.03 ± 0.48	NIST Library
32.389	methyl 9-oxononanoate	1931-63-1	2082	1436	-	2.93 ± 1.88	8.45 ± 3.68	12.73 ± 0.63	14.84 ± 1.95	32.43 ± 5.86	[43]

Note: “-” indicates that the substance has not been detected.

**Table 3:** Volatile components and content changes in MLO under 180 °C oxidation conditions.

RT	Name	CAS	Actual RI	Theoretical RI	Content (μg/g)						References
					0 h	1 h	2 h	4 h	8 h	12 h	
8.706	Decane	124-18-5	1060	1000	7.57 ± 0.25	6.34 ± 0.41	5.28 ± 0.44	6.21 ± 0.51	6.60 ± 1.37	-	NIST Library
9.684	Hexanal	66-25-1	1110	1110	-	66.82 ± 3.84	100.30 ± 2.42	115.63 ± 3.38	137.83 ± 5.91	160.42 ± 5.86	[33]
10.714	Undecane	1120-21-4	1164	1100	6.54 ± 0.13	6.57 ± 0.42	9.74 ± 0.23	5.34 ± 0.71	5.10 ± 0.14	5.60 ± 0.66	NIST Library
10.783	2-Butylfuran	4466-24-4	1168	1123	-	-	-	-	8.17 ± 1.01	11.54 ± 0.97	[44]
11.607	2-Heptanone	110-43-0	1211	1213	-	-	-	19.71 ± 3.38	22.33 ± 3.22	20.52 ± 0.49	[45]
11.755	Methyl hexoate	106-70-7	1219	1208	-	-	38.46 ± 1.13	56.57 ± 2.64	61.88 ± 5.47	66.72 ± 2.50	[46]
12.144	Trans-2-Hexenal	6728-26-3	1242	1247	-	24.82 ± 0.71	29.66 ± 1.23	33.59 ± 0.71	33.18 ± 1.10	33.30 ± 1.01	[47]
12.459	pentanol	71-41-0	1260	1260	-	32.06 ± 4.29	34.62 ± 11.81	38.68 ± 1.26	45.53 ± 1.17	-	[48]
12.694	2-Amylfuran	3777-69-3	1273	1261	-	105.10 ± 8.18	126.24 ± 5.94	426.16 ± 10.27	750.35 ± 78.37	1063.53 ± 41.40	[49]
13.026	3-Octanone	106-68-3	1292	1284.7	-	-	-	14.87 ± 1.90	25.07 ± 0.62	25.58 ± 0.87	[36]
13.1	3-dodecene	7206-14-6	1296	1237	-	-	-	16.83 ± 2.72	27.65 ± 1.04	37.66 ± 2.34	[50]
13.632	Methyl heptanoate	106-73-0	1326	1302	-	87.48 ± 0.75	114.75 ± 7.82	223.79 ± 7.03	297.36 ± 18.89	343.64 ± 3.52	[51]
13.764	(z)-1,1-diethoxy-3-hexene	73545-18-3	1334	1272	-	9.54 ± 0.89	10.23 ± 0.79	20.59 ± 0.66	-	-	NIST Library
14.113	Heptenal	18829-55-5	1354	1342	6.78 ± 1.10	398.25 ± 6.82	471.01 ± 4.43	545.89 ± 8.76	560.60 ± 8.03	568.92 ± 10.64	[52]
15.624	Methyl octylate	111-11-5	1437	1437	214.40 ± 3.64	1723.38 ± 19.85	2482.75 ± 11.93	3183.01 ± 111.91	3976.52 ± 51.04	4509.02 ± 319.78	[35]
16.207	(2E)-2-Octenal	2548-87-0	1467	1467	-	137.32 ± 2.92	175.45 ± 0.24	357.51 ± 26.60	467.40 ± 13.88	511.40 ± 23.08	[36]
16.264	Trans-2-Octen-1-ol	18409-17-1	1470	1470	-	42.75 ± 2.44	68.70 ± 5.71	-	-	-	[37]
16.465	methyl (Z)-4-octenoate	21063-71-8	1481	1481	-	16.46 ± 0.40	33.12 ± 2.87	49.69 ± 8.52	81.51 ± 5.68	102.39 ± 2.84	[53]
17.809	Methyl nonanoate	1731-84-6	1544	1536	-	13.54 ± 1.05	21.94 ± 0.03	52.14 ± 2.36	96.58 ± 9.41	145.53 ± 4.48	[35]
17.947	(E)-3-nonen-2-one	18402-83-0	1551	1523	-	19.59 ± 0.37	25.12 ± 3.93	39.89 ± 1.83	50.35 ± 1.68	53.31 ± 1.12	[54]
18.342	Amyl hexanoate	540-07-8	1569	1525	-	-	-	-	30.43 ± 5.85	43.64 ± 2.12	[55]
18.576	(2E)-2-Nonenal	18829-56-6	1579	1574	-	10.28 ± 2.24	12.18 ± 2.14	27.22 ± 2.27	50.59 ± 6.95	90.72 ± 5.26	[39]
18.811	methyl non-8-enoate	20731-23-1	1590	1216	-	39.31 ± 2.15	51.08 ± 0.95	132.90 ± 3.13	242.29 ± 27.04	338.94 ± 23.28	[56]
21.46	methyl dec-4-enoate	1191-02-2	1699	1622	32.37 ± 1.29	67.34 ± 0.66	86.51 ± 8.20	150.70 ± 16.88	255.62 ± 32.40	321.20 ± 13.62	[40]
22.461	(2E,4E)-2,4-Nonadienal	5910-87-2	1737	1738	-	26.03 ± 3.65	33.16 ± 2.47	47.41 ± 7.33	85.37 ± 8.60	95.70 ± 15.13	[42]
24.75	Methyl undecenate	111-81-9	1822	1718	-	16.85 ± 0.85	27.63 ± 1.78	43.87 ± 7.44	69.06 ± 9.37	82.94 ± 4.83	[40]
25.511	(2E,4E)-Deca-2,4-dienal	25152-84-5	1849	1852	66.31 ± 4.92	884.87 ± 40.47	1380.79 ± 91.85	1634.30 ± 321.65	1984.50 ± 10.00	2207.03 ± 112.83	[42]
26.112	methyl 10-methylundecanoate	5129-56-6	1870	1472.4	-	9.35 ± 0.38	12.50 ± 1.33	12.81 ± 0.11	12.08 ± 3.13	11.64 ± 0.38	NIST Library
6.124	Methyl laurate	111-82-0	1871	1834	10.06 ± 1.08	-	-	-	-	-	[35]
28.99	methyl 8-oxooctanoate	3884-92-2	1963	1334	-	42.79 ± 1.63	76.8 ± 8.65	212.88 ± 14.91	438.96 ± 109.59	536.68 ± 52.91	NIST Library
32.389	methyl 9-oxononanoate	1931-63-1	2082	1436	-	83.94 ± 9.15	159.50 ± 14.13	360.94 ± 16.77	679.81 ± 168.76	939.94 ± 31.63	[43]
32.95	methyl 8-hydroxyoctanoate	20257-95-8	2104	1326	-	-	5.57 ± 0.58	44.88 ± 0.48	126.66 ± 24.07	305.03 ± 1.69	NIST Library
35.159	2-octyl furan	4179-38-8	2232	1530	-	-	-	83.45 ± 12.28	246.30 ± 87.77	513.34 ± 19.69	NIST Library

Note: “-” indicates that the substance has not been detected.

**Table 4:** Lipophilic aldehydes produced by MLO oxidation at 180 ° C for 12 h.

Number	RT	Name	Molecular Formula	Mass-to-Charge Ratio After Derivatization (*m*/*z*)
1	1.23	Hydroxy-butanal	C_4_H_8_O_2_	267
2	2.07	10-Oxo-8-decenoic acid	C_10_H_16_O_3_	363
3	4.41	9-Hydroxy-12-oxo-10-dodecenoic acid	C_12_H_20_O_4_	407
4	4.55	6-Hydroxy-heptanal	C_7_H_14_O_2_	309
5	4.98	Valeraldehyde *	C_5_H_10_O	265
6	5.05	(2E)-4-Hydroxy-2-nonenal *	C_9_H_16_O_2_	335
7	5.07	4,7-Dihydroxy-heptanal	C_7_H_12_O_3_	323
8	5.27	9-Oxo-octanoic methyl ester	C_11_H_20_O_3_	365
9	5.48	Hexanal *	C_6_H_12_O	279
10	5.52	Oxo-undecenoic acid	C_11_H_18_O_3_	377
11	5.73	Heptenal	C_7_H_12_O	291
12	6.01	(2E)-2-Octenal *	C_8_H_14_O	305
13	6.06	(2E,4E)-2,4-Nonadienal	C_9_H_14_O	317
14	6.24	(2E)-2-Nonenal	C_9_H_16_O	319
15	6.31	(2E,4E)-Deca-2,4-dienal *	C_10_H_16_O	331

Note: * represents has been compared with the standard.

**Table 5:** PLSR calibration and cross-validation results of inflammatory gene expression levels under 60 °C oxidation conditions.

	Calibration		Cross-Validation		
	RMSEC	R^2^_C_	RMSECV	R^2^_CV_	RPD_CV_
TNF-α	0.061	0.961	0.125	0.864	2.599
IL-1β	0.050	0.976	0.085	0.942	3.966
COX-2	0.027	0.928	0.059	0.703	1.743

**Table 6:** PLSR calibration and cross-validation results of inflammatory gene expression levels under 180 °C oxidation conditions.

	Calibration		Cross-Validation		
	RMSEC	R^2^_C_	RMSECV	R^2^_CV_	RPD_CV_
TNF-α	0.079	0.940	0.104	0.912	3.230
IL-1β	0.191	0.962	0.250	0.945	4.079
COX-2	0.076	0.942	0.099	0.917	3.330

## Data Availability

The original contributions presented in this study are included in the article/Supplementary Material. Further inquiries can be directed to the corresponding author.

## Supplementary Materials

The following supporting information can be downloaded at: https://www.mdpi.com/article/10.3390/foods14244231/s1, Figure S1: Effects of different concentrations of oxidized MLO on the viability of RAW264.7 cells. A-60 °C; B-180 °C; Figure S2: Effects of oxidized MLO on the mRNA expression of three inflammatory cytokines under different incubation concentrations and times. A-60 °C; B-180 °C; Table S1: Standard working curves of 5 aldehydes.
