# Supplementary material for: Using Volatile Oxidation Products to Predict the Inflammatory Capacity of Oxidized Methyl Linoleate
Source: Foods. 2025 Dec 9;14(24):4231. doi: 10.3390/foods14244231 (PMC12731653; doi:10.3390/foods14244231)
Supplement: Supplementary file 1 [file foods-14-04231-s001.zip › foods-4010495-supplementary.pdf]

Table S1. Standard working curves of 5 aldehydes

Aldehyde compounds	RT	Standard curve formula	R2
Valeraldehyde	4.98	y=2492.3x+3250.4	0.9903
Hexanal	5.48	y=2145.9x+667.5	0.9968
Heptenal	6.01	y=23068x+281.93	0.9994
(2E,4E)-Deca-2,4-dienal	6.31	y=53276x-2748.2	0.9992
(2E)-4-Hydroxy-2-nonenal	5.05	y=8708.3x+561.75	0.9921

Note: The contents of other lipophilic aldehydes were calculated using the HNE standard curve formula.

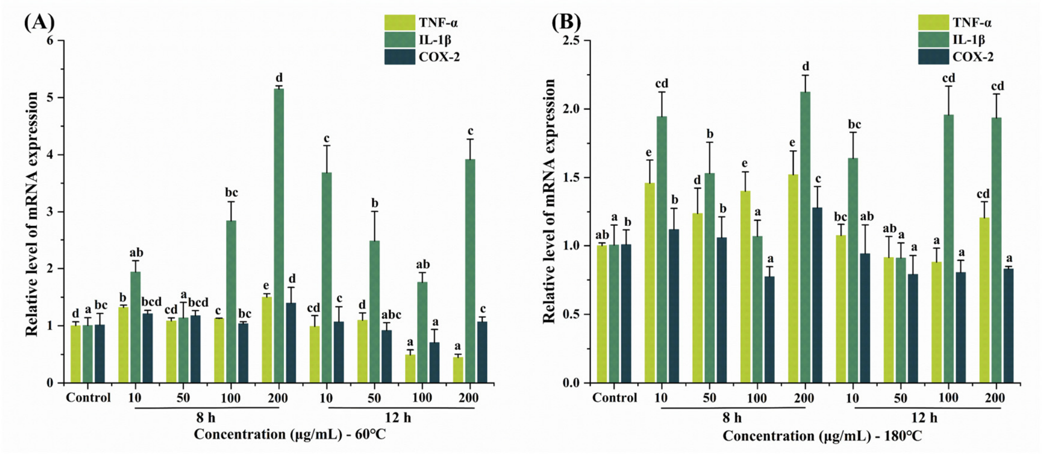

Figure S1. Effects of different concentrations of oxidized MLO on the viability of RAW264.7 cells. A—60°C; B—180°C

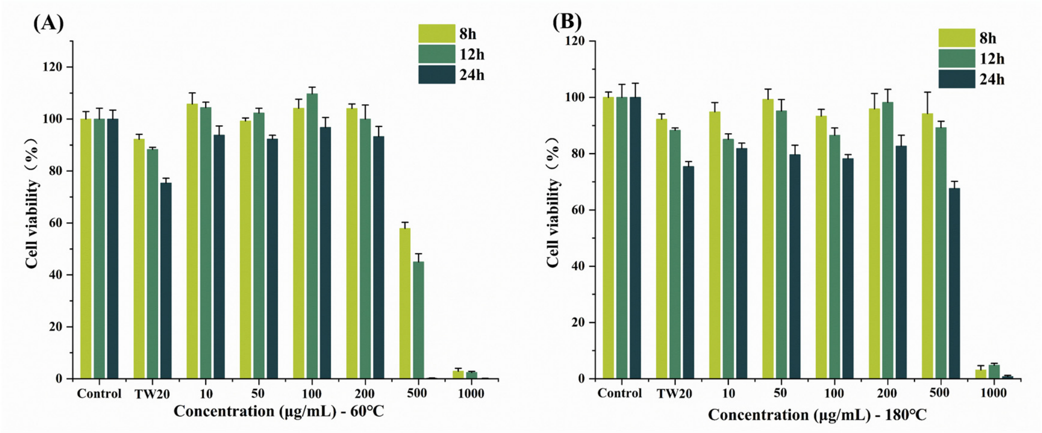

Figure S2. Effects of oxidized MLO on the mRNA expression of three inflammatory cytokines under different incubation concentrations and times. A—60°C; B—180 °C
